# Why high tech needs high touch: Supporting continuity of community primary health care

**DOI:** 10.4102/phcfm.v10i1.1616

**Published:** 2018-06-21

**Authors:** Ellenore D. Meyer, Johannes F.M. Hugo, Tessa S. Marcus, Rebaone Molebatsi, Kabelo Komana

**Affiliations:** 1 Department of Family Medicine, University of Pretoria, South Africa; 2 Department of Obstetrics and Gynaecology, School of Medicine, University of Pretoria, South Africa

## Abstract

**Background:**

Integrated care through community-oriented primary care (COPC) deployed through municipal teams of community health workers (CHWs) has been part of health reform in South Africa since 2011. The role of COPC and integration of information and communication technology (ICT) information to improve patient health and access to care, require a better understanding of patient social behaviour.

**Aim:**

The study sought to understand how COPC with CHWs visiting households offering health education can support antenatal follow-up and what the barriers for access to care would be.

**Method:**

A mixed methodological approach was followed. Quantitative patient data were recorded on an electronic health record-keeping system. Qualitative data collection was performed through interviews of the COPC teams at seven health posts in Mamelodi and telephonic patient interviews. Interviews were analysed according to themes and summarised as barriers to access care from a social and community perspective.

**Results:**

An integrated COPC approach increased the number of traceable pregnant women followed up at home from 2016 – 2017. Wrong addresses or personal identification were given at the clinic because of fear of being denied care. Allocating patients correctly to a ward-based outreach team (WBOT) proved to be a challenge as many patients did not know their street address.

**Conclusion:**

Patient health data available to a health worker on a smartphone as part of COPC improve patient traceability and follow-up at home making timely referral possible. Health system developments that support patient care on community level could strengthen patient health access and overall health.

## Introduction

Over the last decade, the South African Health Department has prepared for major health reforms to address the inefficiencies of a two-tiered system where the private sector spends equal amounts on health care in comparison with the public sector, but less than 20% of the population is able to access private care.^1^ In its efforts to address inequality and issues with access to care, especially for people in informal settlements with low access to care, the Department of Health (DoH) proposed that it would incrementally work towards a National Health Insurance and implement major changes in health care delivery, starting with primary care.^2^ In 2010, the South African Government revised its strategic framework goals over the medium term and set 12 outcomes to achieve this.^3^ The DoH took responsibility for Objective 2: ‘a long and healthy life for all South Africans’.^4^ In response to this, the DoH set four outputs to achieve,^3^ (1) increasing life expectancy, (2) decreasing maternal and childhood mortality, (3) improving HIV and/or AIDS morbidity and mortality and (4) strengthening the health system’s effectiveness.

The re-engineering of primary care led to the adoption of community-oriented primary care (COPC), a municipal ward health system, implemented in 2011, and the employment of teams of community health workers (CHWs) each assigned 150 – 200 households in a municipal ward.^5,6^ Community health workers visit each household and offer basic health education and services, and refer appropriately to the nearest primary care facility. The CHWs are teamed geographically and managed by a team leader who has a health background (typically a nurse).

The University of Pretoria, Department of Family Medicine, partnered with specialists in information technology and developed a health application that CHWs in the Tshwane region have been using to capture household data on smart phones since 2011.^7^ A year and a half later, mid-2012, over 40 000 individual health status assessments had been captured electronically and could be utilised to analyse and plan future developments.^6^ The lean start-up approach was initiated with the focus first on gathering health and social data of households that could inform strategic health interventions on ward, community and regional level. Follow-up modules that target specific needs, for example, antenatal care, could then be developed.

The concept of COPC is as old as the early 1940s when Sidney Kark piloted such a model in South Africa.^6^ In the early 1980s, there was a renewed focus on such a model, and the terminology ‘COPC’ arose out of an international workshop hosted by the Institute of Medicine which advocated for health professionals to be trained in the principles of community-oriented primary care.^8^ The Institute of Medicine’s definition of COPC has three distinct principles^8^, (1) it is rooted in primary care that is accessible, comprehensive, accountable and continuous, (2) It is context and community specific and incorporates schools, religious and other relevant community health supporting structures, (3) It defines a community geographically or based on other distinct traits, conducts a diagnosis for such a community, appropriately plans, intervenes and analyses its effectiveness and adjusts accordingly in participation with the local members. The ward-based outreach team (WBOT) approach with CHWs assigned to a number of households in geographically defined municipal wards was adopted by the National Health Council in November 2010.^8^ Various models of deployment of health services through WBOT were piloted throughout the country. The University of Pretoria in partnership with the DoH and other organisations implemented a COPC model which the current dean of the University of Pretoria coined ‘Kark on crack’, because the model drew from the historic roots of COPC and primary care as piloted by Sidney Kark in the 1940s, but it also used the latest applications of technology, such as smartphones, to gather health data on individuals and families to diagnose and inform future health and social strategies and resource allocation on local municipal, but potentially, national level.

Community-oriented primary care, a ‘high tech, high touch’ approach, with care being delivered in citizens’ homes and with the distribution of this service being based on equity to reach the poor and vulnerable first, was seen as a vehicle for the DoH to achieve the four outputs it set for itself in 2010.^3^

The initial successes of COPC and the possibility of future health preventative and intervention modalities led to numerous partnerships for explorative research, such as linking COPC and home-based health service delivery that is technologically driven (linking home and clinical facility data between health care providers to optimise care, using smartphones) with antenatal Doppler (Umbiflow) in a public sector primary care facility.^9^ Community-oriented primary care and home-based follow-up to screen for other health and social issues and to ensure timely follow-up of the mother and child promised benefits for such an intervention. The theory made sense; the question was whether in practice, COPC and personalised home-based antenatal care could impact on the choices and health of mothers (and foetuses) at risk.

## Key focus

This article seeks to understand how antenatal care users in a public facility in South Africa access care: the benefits and challenges of linking them to a CHW on household level through the use of AitaHealth^TM^. It also explores the obstacles and opportunities of using technology in a primary and community care setting and the learning experience in practice to ensure successful implementation.

## Objective

Firstly, the objective was to understand how household to clinical facility-integrated COPC, using ICT and working with CHWs, can effectively be combined with routine clinical antenatal care in improving antenatal, postnatal, and maternal and child care. Secondly, the objective was to determine whether follow-up of patients on household level, through the principles of COPC, could improve patient traceability and ultimately patient support to ensure antenatal attendance at a clinical facility.

## Contribution to the field

Community-oriented primary care, through health services offered to families in municipal wards by CHWs, is an important component of health re-engineering in South Africa.^5^ More so, if government wants to implement a National Health Insurance successfully, it has to understand the migratory and other social patterns that influence accessing health care. This article offers insight on how the use of integrated ICT informs on patients’ mobility, even during times of having a need for a specific health service, and the impact it has on their access to care and inevitably their (and the foetus’) health. It offers health professionals, the government and non-governmental organisations insights into the challenges and opportunities of creating integration of care between the health service site (clinic) and the home.

## Research methods and design

### Design

Pregnant women seen at the Stanza Bopape health care facility for antenatal care (ANC) were registered electronically and their addresses used to assign a WBOT and a CHW to follow up the patients at home to support the ANC throughout the pregnancy. Patients were seen over a one-and-a-half-year period (2016 –2017). At the start of 2016, CHWs started doing household visits and patients were traced via telephone calls according to the COPC approach. The study was interrupted in mid-2017 because of employment problems at municipal level. The available data over the period totalled 1846 patients (1145 for 2016 and 701 for 2017). The impact of COPC and reasons for failure to reach patients were analysed based on the data. Women with high-risk pregnancies, as identified with the Umbiflow Doppler, were phoned by research assistants, as part of the COPC team’s household visits, to motivate regular follow-up at the clinic and secondary facility. The research assistants were from a social and environmental background and worked as a team to interview the CHWs and patients.

### Materials

Prior to data gathering and interviewing at the clinic registration desk on the electronic record system as well as conducting of the Doppler test, information on patient flow and antenatal care at the Stanza Bopape clinic was obtained through interviews with the clinical staff and management. A computer with internet was installed for interviews at the facility in the waiting area, and a second laptop was used during interviews of patients in an examination room during the Doppler examination. The data were summarised on an Excel spreadsheet according to municipal health posts and given to the WBOT team leaders weekly to follow up on household level. Individual patients were phoned if they were not found at home. Interviews were conducted with WBOT team leaders and CHWs, and also with patients either telephonically or in person when they presented at the facility for an antenatal care visit.

### Procedures

Antenatal follow-up in the public sector often ensues very late in pregnancy with many patients only booking a first visit late in the second trimester. The Umbiflow Doppler research targeted women between 28 – 32 weeks as from then on, a pregnancy was considered viable and early interventions (such as an early delivery via caesarean section) could be performed in the case of a high-risk pregnancy where the baby could be at risk of intrauterine death because of the lack of end diastolic flow. The resistance index was measured and expressed as a numeric value that categorised patients either as a high or low-risk patient. Patients whom presented at the clinic and were between the gestational criteria were selected for follow-up visits at home, although all the patients seen at the clinic would at some stage receive a home visit by a CHW.

Patients seen at Stanza Bopape clinic’s ANC facility were recorded electronically on Synaxon. The patient’s demographics, specifically the address within a municipal designated ward, was used to assign the patient to a WBOT and to inform the team leader. At the WBOT health post, a patient was allocated to a specific CHW by the team leader depending on the street address. Community health workers were motivated to visit the patient as early as possible. A household registration was done electronically on AitaHealth^TM^. The patients were followed up four to five times throughout their pregnancy at the ANC Clinic, whilst a CHW visited the patients at home between clinic visits. High-risk patients received special attention with CHW visits at home within a week of being seen at the clinic, whilst patients considered to have a normal pregnancy were followed up within a month.

The COPC approach and regular follow-up of patients at home raised an awareness that patients were not always found at the address provided at the clinic, even when CHWs did home visits very soon after registration. This prompted the investigation and telephonic calls to search for patients to understand the reasons behind this and search for ways to increase follow-up at home. Patients who were phoned for interviews were reminded of their previous visit and their consent given at the clinic and were again asked to give consent via telephone to a short interview. The telephonic interviews were conducted by a doctor associated with the University of Pretoria in English and if the patient did not understand, interpreted by a research assistant.

Wherever possible, the patient interviews and data gathering were conducted in both English and the patient’s mother tongue. Written patient consent was obtained after an information leaflet was handed to patients, and an explanatory session was conducted by the health professional doing the Doppler. Prior to registration on the electronic record-keeping system and Umbiflow Doppler, the patients signed an informed consent document for sharing of information with a WBOT and CHW.

The COPC research ran until June 2017 because of interruption of CHW contracts from the local municipality that employed them. During 2016, because of the high number of patients not traceable or supplying an incorrect address, a number of interventions were undertaken to improve the patient follow-up. These interventions included training of personnel at Stanza Bopape clinic on the importance of patient education of WBOT visits, improving traceability and follow-up at the clinic by capturing a correct patient address and contact details, training of CHWs on early household visits after a patient had first been seen at the clinic and post-clinic visit follow-ups of high-risk patients via phone calls.

The secondary facility and obstetrician involved in the study had monthly meetings with the research team to compare patient follow-up at facility level and home visits, to identify patients at risk in need of follow-up at home and to compare patient details given to increase traceability.

### Analysis

All interviews and patient records were typed electronically either on the Synaxon electronic health record or on an Excel spreadsheet by a health professional or data administrator part of the Umbiflow and/or COPC team and on the AitaHealth^TM^ app on a smartphone used by CHWs during household visits. Telephonic conversations with patients were summarised, with other relevant details, against the patient’s name on an Excel spreadsheet, and notes were made with every household visit and phone call. Patient data were summarised and checked weekly. No electronic recordings were made of interviews as various languages with translators were used throughout.

## Ethical considerations

This research was approved by the Faculty of Health Sciences, University of Pretoria, protocol number (102/2011) for COPC and also for Umbiflow protocol number (473/2014). The Gauteng Province DoH and Tshwane district Office also gave ethical approval to conduct the research.

## Results

In 2016, a total number of 1145 patients met the gestational criteria for Doppler (28–32 weeks). Of these patients, 39.4% (451) were traceable either by household visit or by interview via a phone call; 17.8% (204) patients had supplied an incorrect address or an address where they did not reside anymore at the time of the follow-up home visit, but were still reachable via phone. Of all the patients seen in 2016, 18.1% (207) were completely untraceable, having given an incorrect address as revealed by CHW home visits and an incorrect phone number. Some patients were not seen by the COPC team because of high patient demand; 24.7% (283) were lost to follow-up at home (not visited by a CHW and unreachable on the contact number supplied).

**FIGURE 1:**
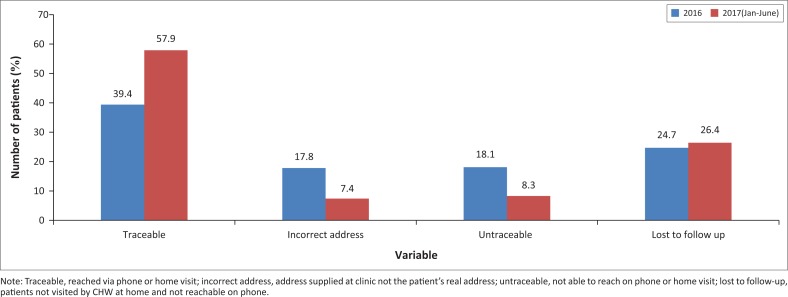
Community-oriented primary care visits for antenatal care patients at Stanza Bopape – Patient follow-up 2016-2017 through WBOTs.

During January – June 2017, a total number of 701 patients were seen. The patients that were traceable, either by household visit or by phone call, had increased to 57.9% (406). Of the remaining patients, 7.4% (52) had supplied an incorrect address or an address where they did not reside anymore at the time of the follow-up home visit. However, only 8.3% (58) of the patients were completely untraceable, having given an incorrect address and phone number, resulting in a 10% decrease. The number of patients lost to follow-up (not visited by CHW and unreachable via phone) increased slightly, being 26.4% (185) for 2017. Figure 1 summarises the improvement in traceability of patients from 2016–2017.

The following barriers to access care at home were identified:

### Communication

Patient interviews via phone revealed various communication issues, such as patients often sharing a cell phone with a family member. When phoning a patient, a boyfriend or other relative would answer the phone. From time to time, the person answering the phone would initially deny knowing the person or ask for incentive to give information about such a person. Reluctance and suspicion of the worthiness of sharing information on the patient made it difficult to contact the patient.

Communication was also difficult as many patients spoke poor English and a translator had to be used. Some patients only spoke a language from a neighbouring country, and thus, communication was very limited.

### Residential factors

When patients were visited but not found at home, a neighbour or family member, if present, was asked to confirm whether the patient was staying at the address supplied. Patients not found at home at the time of the first visit were often reported, by interviews with the CHWs or patients via phone, to be at work, as CHWs do household visits during the week and in the morning up until 14:00 in the afternoon.

Patients who were found not to be living at the address supplied to the clinic were phoned and interviews revealed that they either were not staying there anymore or had never stayed at the given address. Some patients interviewed via phone admitted that they ‘bought’ or supplied an address that falls under the geographical referral area of the clinic as they were afraid they would be turned away if they did not supply an address and proof of residence of such a nature.

Other patients came to stay with someone in the area to have an ANC visit and then returned to another province or neighbouring country to have their baby born there: this remained unchanged over the whole research period (2016 – 2017). During interviews, patients were found to have travelled in-between their home countries, including Zambia, Mozambique and Zimbabwe, amongst others, and only planned to return just before their estimated date of delivery.

Another residential issue was the absence of proper street names or numbers in the informal settlements. Patients therefore did not know their address or struggled to accurately supply an address. Community health workers also struggled to identify the correct home because of this.

### Transport issues

The primary clinic, Stanza Bopape, was within walking distance for over 90% of the patients, as almost everyone supplied an address within a five km radius of the clinic, though not necessarily the correct address. It could therefore be assumed that they could more easily attend their visits there. High-risk patients that were referred to the neighbouring secondary facility to see an obstetrician sometimes did not attend their appointment and reported to a CHW not having had money at the time of the appointment to get a taxi and very few patients had access to a personal car for transport.

## Discussion

The COPC intervention through WBOTs where CHWs follow up antenatal patients at home highlighted difficulties that patients face to access care on household level. The information obtained through home visits and telephonic interviews enabled the team during 2016 to adjust its approach to improve patient support towards health on household level. The improvement of patient visits from 2016 – 2017 indicates that the COPC approach linked to clinical care on facility level should be explored in more studies and patient care settings in future. If one wants to incorporate high tech through electronic record-keeping of patient data and communication and health education via phone or other technological aids, then it needs to be balanced with high touch where trust and good communication between a health provider and patient enable capturing of accurate contact details and patients feel safe to share the barriers they experience to access care.

The plight of the patient to access care needs greater exploration. Interviews with patients during the study offered greater insights into the community-based factors that reiterate what was previously reported on by the DoH as contributors to maternal and foetal deaths.

## Limitations of the study

The limitations of the study would be in the population group sampled. The community exposed to the integrated care and COPC were all accessing one ANC clinic for care. Even though it is probable that most communities living in informal settlements in South Africa also have large percentages of ‘in-transit’ members, that is, people continuously moving across provinces and borders, it cannot be generalised to all residents and individuals that access health services.

## Recommendations

The study or similar health studies should be repeated or expanded to other towns and provinces in South Africa. To design a health system that is high touch and high tech and meets the needs of our patients and achieve the National Department of Health (NDoH) goals, one needs to further explore the socio-economic barriers to accessing care in the South African environment.

## Conclusion

The follow-up of patients at home, with WBOTs through COPC, strengthened by the use of SMSes or phone calls to trace patients not found at home or not coming for follow-ups, could improve access to care, encourage adherence to treatment and help with early identification of social and other health issues on household level, which makes timely referral possible. This should be balanced with the cost of employing CHWs and the budgeting for the smart phone devices, airtime for sending electronic messages and utilisation of an electronic health record system. Without it, patients that are lost to follow-up or who travel across borders or display delayed help-seeking behaviour are not identifiable, and the record of the reasons is not necessarily available to the next health professional that they encounter.

The South African environment, especially that of the vulnerable and the poor living in informal settlements, is often that of a community in-transit. If the health system wants to effectively target and assist these patients to achieve better health for all, then the system which includes patient contact, linked with affordable technology, needs to be designed to meet the needs of our patients and trace and assist them to access care across provinces and perhaps even across borders.
